# Sex differences in prevalence and risk factors of hypertension in India: Evidence from the National Family Health Survey-4

**DOI:** 10.1371/journal.pone.0247956

**Published:** 2021-04-13

**Authors:** Krishna Kumar, Sheuli Misra

**Affiliations:** Centre for the Study of Regional Development, School of Social Sciences, Jawaharlal Nehru University, New Delhi, India; Institute of Economic Growth, INDIA

## Abstract

To estimate sex-specific prevalence and associated socio-economic, demographic, and lifestyle risk factors of hypertension in India. We used data from the National Family Health Survey (NFHS-4) of 2015–16. The analysis based on 6,99,686 women (15–49 years) and 1,12,122 men (15–54 years) whose blood pressure (BP) were measured during the survey. Bivariate distribution was used to show the prevalence of hypertension and, maps were used to present its spatial patterns. Logistic regression model was used to identify sex-specific association between risk factors and hypertension. Results show that the overall prevalence of hypertension was 16.32% among men and 11.56% among women. We also found that the prevalence of hypertension across selected socio-economic, demographic and lifestyle background characteristics and in a majority of the states was higher among men compared to women. Odds ratios from logistic regression analysis direct sex-related differences in risk factors. Hypertension increases with an increase in age and the risk is higher among older women (AOR, 5.58; 95% CI, 5.16–6.03 for women aged 40–49 and AOR, 4.24; 95% CI, 3.94–4.57 for men aged 50–54) compared to men. Education, types of jobs (specially technical, administrative and managerial), marital status and non-vegetarian diet were significantly associated with hypertension in men. While other than age; non-working, consumption of alcohol, and being a diabetic was found to be major risk factors for this disease among women. There are sex-related differences in prevalence as well as risk factors of hypertension in India. In order to prevent early developments of hypertension, awareness related to changing lifestyles such as a diet rich in fruits, vegetables as well as screening to control BP should be promoted among youths and adults in India. The study also recommends sex-specific approaches in health infrastructure and policies besides increasing public awareness.

## Introduction

Hypertension or high blood pressure (hereafter BP) as one of the leading risk factors of global disease burden has contributed to a considerable number of premature deaths worldwide [1]. It has become a major public health problem as 1 in 4 men, and 1 in 5 women had hypertension worldwide in 2015 [2]. Hypertension is a form of the cardiovascular disorder that results from a wide range of interconnected aetiologies [3]. It is also one of the leading risk factors for chronic heart disease, ischemic heart disease, and stroke. About 54% stroke and 47% ischemic heart disease worldwide were attributed to high BP [4]. Furthermore, untreated and uncontrolled hypertension leads to structural and functional abnormalities of the cardiovascular system and harm other vital organs of the body such as kidney, and brain resulting kidney failure, blindness, rupture of blood vessels and cognitive impairment [5].

Recent estimates suggest that the prevalence of hypertension is increasing like an epidemic in many low and middle-income countries (LMICs), while prevalence is static in high-income countries [6,7]. An estimated 1.13 billion people worldwide had hypertension and two-third of them living in low and middle-income countries in 2015 [2]. Hypertension and related complications are a major contributor to death and disability in South Asian countries like India, Bangladesh, Nepal, Bhutan, and Sri Lanka [8]. A study documented that prevalence of hypertension in India had a three-fold increase between 2004–05 and 2011–12 which was more than WHO’s Southeast Asia’s regional level (25%) [9]. In India, hypertension accounts for 5.1% of total deaths and as a leading risk factors constitutes 15% of all deaths due to cardiovascular diseases [10].

Previous studies have identified socio-economic status, family history, geographic heterogeneity, dietary pattern, behavioral factors, obesity, and age as some of the major risk factors associated with hypertension [11–16]. However, studies on hypertension are limited in India due to lack of regular data availability, and few previous studies are done on a small scale based on small sample size or along with other non-communicable diseases [6,9,17–20]. A recent study has provided estimates on the prevalence of hypertension up to district level [21]. So far in our knowledge, no research has tried to investigate sex-specific risk factors of hypertension and how their effects differ for men and women in India. The present study expects that differences in biological as well as modifiable behavioral factors may affect prevalence and incidence of hypertension across sex in India. Identifying sex differences in the susceptibility and development of diseases and specific risk factors are of increasing public health importance [22]. Sex differences have been reported for hypertension in several studies conducted both in high and low-middle income countries [23,24]. Differences in biological as well as modifiable behaviors were found to affect vulnerability, prevalence and incidence of hypertension which in long term lead to cardiovascular diseases (CVDs). A recent study among Korean adults showed that sex-specific differences in prevalence and control of prevalence calls for sex-specific approaches of effective BP management [25]. Moreover, SDGs also target reduction of one-third premature mortality from non-communicable diseases through prevention and treatment by 2030. Therefore, this study aims to evaluate the prevalence as well as provide evidences related to major risk factors of hypertension by sex to help formulate effective public health interventions against the development of hypertension at an early age in India.

## Materials and methods

### Data source

We used the country’s one of the important demographic and health database, the latest round of National Family Health Survey 2015–16 (NFHS-4). This multi-round large scale survey was conducted under the leadership of the Ministry of Health and Family Welfare (MoHFW) and coordinated by the International Institute of Population Sciences (IIPS), Mumbai. It provides essential information on household population and housing characteristics, basic demographic and socio-economic characteristics of respondents, fertility, family planning, infant and child mortality, maternal and child health, nutrition and anemia, morbidity and health care, HIV/AIDs including other adult health issues, women empowerment, and domestic violence at the all India, state, and the district levels. However, the NFHS-4 was first in the NFHS series to provide reliable information on emerging health-related issues like blood glucose levels and hypertension in the general population.

### Study design and sample size

With a stratified two-stage sample design, the NFHS-4 was administered across Primary Sampling Units (PSU) in rural areas and Census Enumeration Blocks (CEB) in urban areas. The background characteristics of eligible survey participants were collected using two different structured questionnaires e.g. men and women separately. While, crucial information on BP measurement including information on anemia, HIV prevalence, blood glucose, height, and weight were collected using the ’Biomarker questionnaire’ for women aged 15–49 years (*N* = 6,99,686) and men aged 15–54 years (*N* = 1,12,122). The survey protocol including the content of all survey questionnaires was approved by the IIPS Institutional Review Board and the ICF Institutional Review Board, and reviewed by the U.S. Centers for Disease Control and Prevention (CDC). Further details of the sampling design used for the survey can be found in the national report of NFHS-4 [26].

### Outcome variable

The measurements of BP for each respondent were taken three times with an interval of five minutes using the OMRON BP monitor by a skilled health investigator. Though, the BP level was calculated taking the average of the last two measurements. A person whose average systolic blood pressure (SBP) was greater than or equal to 140 mmHg or average diastolic blood pressure (DBP) was greater than or equal to 90 mmHg or a person who is currently taking prescribed medicine to lower his/her elevated BP was considered to be hypertensive. For analysis, we constructed a dichotomous hypertension variable where samples with hypertension (defined earlier) given code 1 and non-hypertensive as 0.

### Predictor variables

We considered demographic, socio-economic, and lifestyle characteristics as covariates in order to identify the important risk factors associated with hypertension among men and women. Age was categorized as 15–24, 25–39, 40–49 for women and 15–24, 25–39, 40–54 for men, respectively. Other demographic and socio-economic information includes place of residence (rural or urban), education level (illiterate, primary, secondary or higher), marital status (never married or married), religion (Hindus, Muslims or others), caste (SC, ST, OBC or others), wealth quintile (poorest, poorer, middle, richer or richest), and state of residence. The lifestyle factors were frequency of drinking alcohol (yes or no), consumption of non-vegetarian food (yes or no), and types of occupation (not working, professionals, clerical, sales, service, agriculture, or production workers). We also used information on suffering from diabetes (yes or no) to investigate the association of co-morbidity and risk of hypertension by sex.

### Statistical analyses

The prevalence of hypertension (in percentage) among women and men was presented by selected background characteristics. In order to identify individual-level key demographic, socio-economic, and lifestyle factors of hypertension we used multivariate binary logistic regression models. The concept of multifactorial disease like high BP considered to best conceptualized and measured at the individual level [27]. The regression was conducted separately for females and males to investigate whether these risk factors with the prevalence of hypertension differ by sex or not. We applied three models wherein model 1, we controlled for age, place, and state of residence; in model 2, we controlled all demographic and other socio-economic variables, and lastly, in model 3 odds ratios were estimated after adjusting for demographic, socioeconomic as well as lifestyle factors. The estimates from model 1 and model 2 were similar to the pattern observed in model 3 and therefore, it was decided to show the results of model 3 only. The statistical significance was checked with a 2-tailed *P-*value of 0.05 and appropriate sampling weights were used for the analyses. All analysis has been performed using Stata, version 13.1 (StataCorp, College Station, TX). Further, we mapped the state-wise prevalence of hypertension separately for females and males using geographic information system (GIS).

## Results

**Fig 1:**
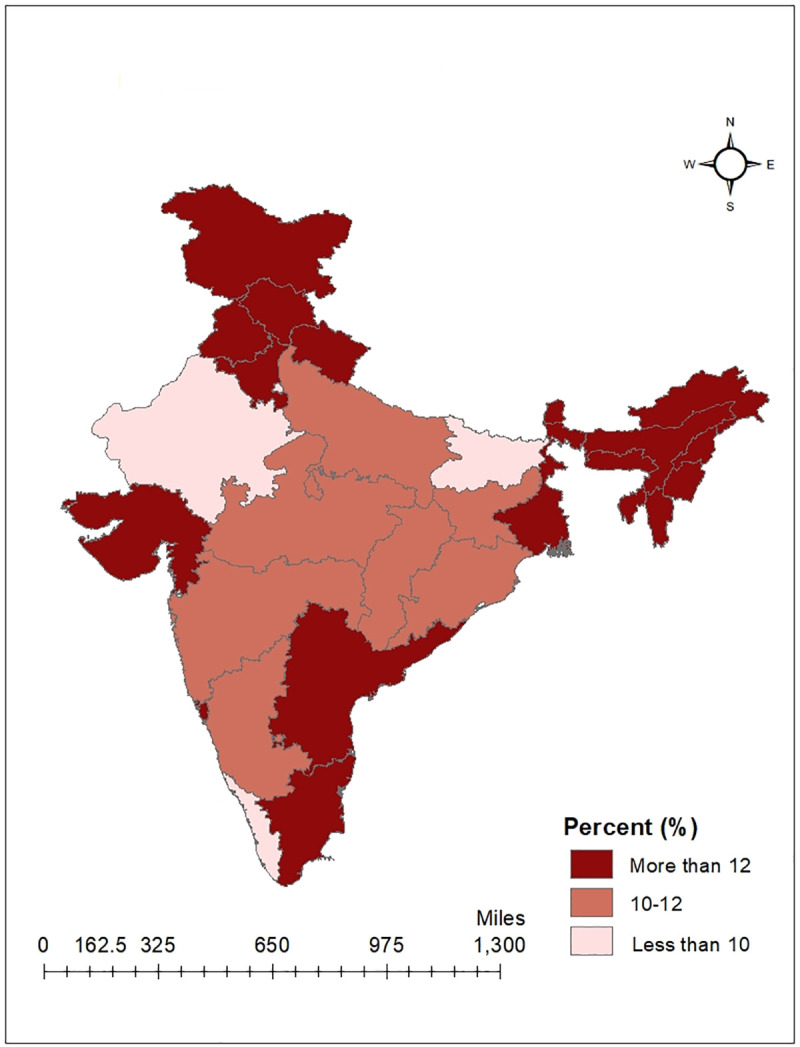
Prevalence of hypertension among women by states of India, 2015–16.

**Fig 2:**
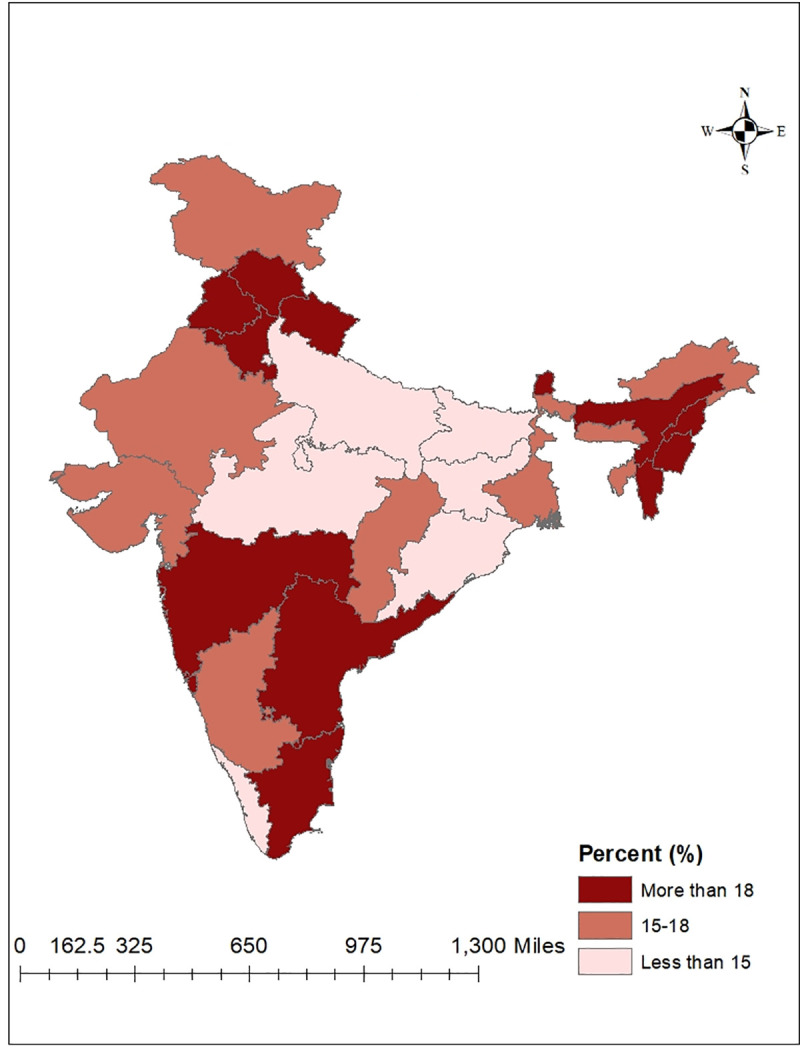
Prevalence of hypertension among men by states of India, 2015–16.

Table 1 shows that overall the prevalence of hypertension among women and men were 11.56% and 16.32%, respectively. Results suggest that the prevalence of hypertension was higher with increasing age. It shows that older adults (40–54 years) had the highest prevalence (24.19% for women and 28.39% for men) and it was nearly double compared to younger age-groups (25–39 years). We found that the prevalence of hypertension was higher among urban residents (12.29% for women and 17.82% for men) compared to their rural counterparts. On one hand, illiterate women were more hypertensive 15.06% and the prevalence was low among higher education level. On the other hand, the prevalence of hypertension was very high among men across all education levels (ranges from 15.07% to 18.83%). Compared to Hindus or Muslims, prevalence of hypertension remained higher for other religious groups (14.00% for women and 19.91% for men). Similarly, other caste groups showed a high prevalence of hypertension (12.55% for women and 17.52% for men) irrespective of sex. Moreover, the prevalence of hypertension risen steadily from poor wealth quintile to the richest wealth quintile (12.87% for women and 19.30% for men) and married people were more hypertensive (13.43% for women and 21.23% for men) than non-married ones. The prevalence by lifestyle characteristics show that adults drinking alcohol almost every day (20.16% for women and 29.26% for men), consuming non-vegetarian diet (12.23% for women and 17.20% for men) and being a diabetic (30.70% for women and 37.17% for men) had higher prevalence of hypertension. However, the prevalence of hypertension by type of occupation found to be high among females engaged in the service sector 13.43% and professional males 23.16% compared to their counterparts. The state-wise prevalence of average BP showed north, north-eastern and southern states had a higher prevalence of hypertension compared to other states of India and prevalence was also higher for males in the majority of states. The highest prevalence of hypertension among men was found in Punjab 25.97%, and for women in Sikkim 18.86%. Meanwhile, Delhi reported the lowest prevalence of hypertensive adults, but women 7.18% were more hypertensive compared to men 5.22%. The state-wise prevalence of hypertension for men and women are presented with two separate maps (Figs 1 and 2).

**Table 1:** Prevalence of hypertension among women and men stratified by socio-economic, demographic and lifestyle background characteristics.

		Women	Men
		(n = 699686)	(n = 112122)
		11.56%	16.32%
**Age group**	15–24	247833 (4.15)	35712 (5.49)
**n (%)**	25–39	302577 (11.20)	44688 (16.24)
	40-49/50-54^a^	149296 (24.19)	31722 (28.34)
**Place of residence**	Urban	204735 (12.29)	35526 (17.82)
**n (%)**	Rural	494951 (11.18)	76596 (15.40)
**State**	Andhra Pradesh	10428 (12.57)	1541 (18.56)
**n (%)**	Arunachal Pradesh	14294 (13.87)	2140 (16.72)
	Assam	28447 (18.64)	4191 (23.22)
	Bihar	45812 (9.30)	5872 (12.57)
	Chandigarh	746 (10.26)	127 (18.03)
	Chhattisgarh	25172 (11.22)	3827 (15.53)
	Goa	1696 (12.15)	848 (18.62)
	Gujarat	22932 (12.29)	6018 (15.98)
	Haryana	21654 (14.06)	3584 (21.88)
	Himachal Pradesh	9929 (14.84)	2417 (24.68)
	Jammu & Kashmir	23800 (16.79)	6013 (17.03)
	Jharkhand	29046 (10.30)	4069 (14.37)
	Karnataka	26291 (11.74)	4106 (17.14)
	Kerala	11033 (9.98)	2086 (13.47)
	Madhya Pradesh	62803 (10.59)	10268 (13.99)
	Maharashtra	29460 (11.81)	4811 (18.39)
	Manipur	13593 (13.28)	1886 (23.78)
	Meghalaya	9202 (14.89)	1236 (15.34)
	Mizoram	12279 (12.78)	1749 (19.02)
	Nagaland	10790 (13.13)	1596 (24.43)
	Delhi	5914 (7.18)	710 (5.22)
	Odisha	33721 (11.65)	4634 (13.55)
	Punjab	19484 (16.29)	3250 (25.97)
	Rajasthan	41965 (9.65)	6309 (15.30)
	Sikkim	5293 (18.86)	879 (32.73)
	Tamil Nadu	28820 (12.20)	5317 (19.92)
	Tripura	4804 (17.01)	878 (17.60)
	Uttar Pradesh	97661 (10.07)	13835 (12.87)
	Uttarakhand	17300 (12.49)	2174 (20.98)
	West Bengal	17668 (12.27)	2645 (15.17)
	Telangana	7567 (13.19)	1133 (19.36)
**Education Level**	Illiterate	196556 (15.06)	15007 (17.81)
**n (%)**	Primary	88290 (13.80)	14351 (18.83)
	Secondary	334927 (9.80)	65260 (15.07)
	Higher	79913 (8.29)	17504 (17.52)
**Religion**	Hindus	519281 (11.22)	83567 (16.40)
**n (%)**	Muslims	94591 (12.57)	15438 (14.05)
	Others	85814 (14.00)	13117 (19.91)
**Caste**	Schedule caste (SC)	124813 (10.83)	19875 (16.26)
**n (%)**	Schedule tribe (ST)	127133 (12.71)	20026 (16.43)
	Other backward caste (OBC)	273700 (11.15)	43434 (15.92)
	Others	141428 (12.55)	22564 (17.52)
**Wealth Quintile**	Poorest	133249 (10.18)	18412 (12.35)
**n (%)**	Poorer	149466 (10.64)	23220 (12.90)
	Middle	147168 (11.12)	24331 (16.12)
	Richer	138502 (12.72)	23383 (18.89)
	Richest	131301 (12.87)	22776 (19.30)
**Marital status**	Never married	171797 (4.20)	40273 (7.39)
**n (%)**	Married	499627 (13.43)	70215 (21.23)
**Frequency of taking alcohol**	Almost every day	2212 (20.16)	4881 (29.26)
**n (%)**	Once in a week	6773 (21.42)	14635 (20.96)
	Less than once in a week	8320 (16.42)	15766 (18.44)
**Suffering from diabetes**	No	679425 (11.20)	108698 (15.86)
**n (%)**	Yes	9538 (30.7)	2150 (37.17)
**Eat non-vegetarian**	No	572059 (11.39)	84184 (15.99)
**n (%)**	Yes	127627 (12.23)	27938 (17.20)
**Types of Job**	Not working	85138 (10.64)	24889 (8.62)
**n (%)**	Professional ^b^	3458 (9.40)	6431 (23.16)
	Clerical	474 (12.45)	2055 (20.85)
	Sales	1874 (12.38)	9847 (21.29)
	Service	4002 (13.43)	7866 (19.93)
	Agriculture	18465 (11.82)	32961 (17.06)
	Production worker ^c^	7534 (11.38)	27863 (17.20)

Source: Author`s calculation from NFHS data.

^a^ The last age-group category for men is 50–54.

^b^ includes technical, administrative and managerial occupations.

^c^ includes skilled and unskilled manual occupations.

In order to investigate which of the demographic, socioeconomic, and lifestyle characteristics were major risk factors of hypertension in India, a logistic regression model was performed. Table 2 shows adjusted odds ratios (AORs) from the regression models for men and women. Results showed that at older ages women had higher odds of hypertension than men (Women: AOR, 5.58; 95% CI: 5.16–6.30; P<0.001; Men: AOR, 4.24; 95% CI, 3.94–4.57; P<0.001). However, higher education was associated with a lower prevalence of hypertension among women (AOR, 0.79; 95% CI, 0.72–0.86; P<0.001) whereas higher educated adult men had an 18% higher risk of hypertension (AOR, 1.18; 95% CI, 1.09–1.27; P<0.001). We found that men engaged in tertiary activities such as professionals, clerical, and sales had a higher risk of hypertension compared to those who were non-working (AOR, 1.16; 95% CI, 1.09–1.24; P<0.001). In contrast, women outside the labor force were more likely to be hypertensive. Incident of hypertension was very high among adults suffering from diabetes. It was two times higher for women (AOR, 2.27; 95% CI,2.02–2.56; P<0.001). Similarly, diabetic men were more likely to be hypertensive, however, their odds (AOR, 1.69; 95% CI, 1.53–1.87; P<0.001) were lower than women.

**Table 2:** Adjusted odds ratios (AORs) for hypertension among women and men, by demographic, socioeconomic and lifestyle characteristics.

Variable		AOR (95% CI)	
		Women	Men
**Age**	15–24	Ref	
	25–39	2.32*** (2.16–2.50)	2.23*** (2.08–2.39)
	40-49/50-54^a^	5.58***(5.16–6.03)	4.24*** (3.94–4.57)
**Place of residence**	Urban	Ref	
	Rural	0.95* (0.90–1.00)	0.97 (0.93–1.01)
**States**	Kerala	Ref	
	Andhra Pradesh	1.35** (1.09–1.68)	1.64*** (1.36–1.99)
	Arunachal Pradesh	1.73*** (1.40–2.13)	1.58*** (1.30–1.91)
	Assam	2.54*** (2.13–3.03)	2.91*** (2.48–3.41)
	Bihar	1.13 (0.95–1.35)	1.29*** (1.10 1.52)
	Chandigarh	1.2 (0.66–2.17)	1.32 (0.80 2.17)
	Chhattisgarh	1.3* (1.08–1.57)	1.56*** (1.32 1.84)
	Goa	1.44* (1.10–1.89)	1.35* (1.07 1.72)
	Gujarat	1.41*** (1.18–1.67)	1.57*** (1.34 1.84)
	Haryana	1.60*** (1.33–1.93)	2.25*** (1.92 2.65)
	Himachal Pradesh	1.76*** (1.46–2.12)	2.39*** (2.02 2.84)
	Jammu and Kashmir	1.81*** (1.52–2.16)	1.78*** (1.50 2.11)
	Jharkhand	1.40*** (1.16–1.68)	1.49*** (1.26 1.76)
	Karnataka	1.37*** (1.14–1.65)	1.86*** (1.58 2.19)
	Madhya Pradesh	1.41*** (1.20–1.67)	1.44***(1.23 1.67)
	Maharashtra	1.45*** (1.21–1.73)	1.85***(1.58 2.17)
	Manipur	1.60*** (1.29–1.98)	2.45*** (2.03–2.96)
	Meghalaya	1.98*** (1.56–2.52)	1.38* (1.10–1.73)
	Mizoram	1.48*** (1.18–1.87)	1.73*** (1.41–2.12)
	Nagaland	2.57*** (2.05–3.21)	2.58*** (2.11–3.15)
	Delhi	0.74 (0.54–1.01)	0.63*** (0.46–0.86)
	Odisha	1.25* (1.05–1.51)	1.11 (0.94–1.31)
	Punjab	1.83*** (1.51–2.20)	2.68*** (2.27–3.16)
	Rajasthan	1.02 (0.86–1.22)	1.53*** (1.31–1.79)
	Sikkim	2.65*** (2.10–3.34)	3.41*** (2.77–4.19)
	Tamil Nadu	1.42*** (1.20–1.69)	1.8*** (1.55–2.11)
	Tripura	1.95*** (1.50–2.53)	1.48*** (1.16–1.88)
	Uttar Pradesh	1.23* (1.05–1.45)	1.34*** (1.15–1.55)
	Uttarakhand	1.18 (0.96–1.44)	2.01*** (1.68–2.40)
	West Bengal	1.49*** (1.22–1.83)	1.53*** (1.27–1.83)
	Telangana	1.41* (1.11–1.78)	1.62*** (1.32–2.00)
**Education level**	Illiterate	Ref	
	Primary	1.04 (0.97–1.11)	1.08* (1.01–1.15)
	Secondary	0.88*** (0.84 0.94)	1.06 (1.00–1.12)
	Higher	0.79*** (0.72–0.86)	1.18*** (1.09–1.27)
**Religion**	Hindu	Ref	
	Muslims	1.22*** (1.14–1.30)	0.99 (0.93–1.05)
	Others	1.01 (0.93–1.10)	0.97 (0.9–1.04)
**Caste**	SC	Ref	
	ST	1.02 (0.94–1.10)	1.07* (1.00–1.15)
	OBC	0.98 (0.92–1.04)	0.95 (0.91–1.00)
	Others	1.08* (1.01–1.16)	1.00 (0.94–1.05)
**Wealth Status**	Poorest	Ref	
	Poorer	1.07 (1.00–1.15)	1.08* (1.02–1.16)
	Middle	1.16*** (1.07–1.25)	1.27*** (1.19–1.35)
	Richer	1.34*** (1.23–1.45)	1.47*** (1.37–1.58)
	Richest	1.36*** (1.24–1.49)	1.56***(1.45–1.69)
**Marital Status**	Never married	Ref	
	Currently married	1.27*** (1.18–1.38)	1.34*** (1.27–1.43)
**Drinking alcohol**	No	Ref	
	Yes	1.38*** (1.23–1.55)	1.26*** (1.21–1.31)
**Suffering from Diabetes**	No	Ref	
	Yes	2.27*** (2.02–2.56)	1.69*** (1.53–1.87)
**Eat Non-veg food**	No	Ref	
	Yes	1.03 (0.97–1.08)	1.05* (1.01–1.10)
**Types of Job**	Not working	Ref	
	Professional^a^	0.92 (0.81–1.05)	1.23*** (1.13–1.34)
	Clerical	0.85 (0.61–1.19)	1.06(0.94–1.20)
	Sales	0.95 (0.81–1.10)	1.16*** (1.07–1.25)
	Service	0.94* (0.88–1.00)	1.00 (0.94–1.07)
	Agriculture	0.99 (0.89–1.11)	1.16*** (1.07–1.25)
	Production worker^b^	0.97 (0.89–1.06)	1.06 (0.99–1.13)

Source: Author`s calculation from NFHS data.

***p value < 0.001

**p value < 0.01

*p value < 0.05.

^a^The last age-group category for men is 50–54.

^b^ includes technical, administrative and managerial occupations.

^c^ includes skilled and unskilled manual occupations.

Among other adjusted factors age, place of residence, marital status, wealth quintile, alcohol, and non-vegetable food intake showed a significant association with the prevalence of hypertension. The prevalence was higher among married men than never-married ones and the odds were greater compared to married women (men: AOR, 1.34; 95% CI, 1.27–1.43; P<0.001; women: AOR, 1.27; 95% CI,1.18–1.38; P<0.001). Prevalence and risk of hypertension was less among surveyed participants from rural areas (men: AOR, 0.97; 95% CI, 0.93–1.01; p = 0.150; women: AOR, 0.94; 95% CI, 0.90–1.00; P = 0.040). Residents of north, north-eastern and southern states except Kerala had higher risk of being hypertensive. The national capital of Delhi also had a lower prevalence of hypertension. Further, the odds were 1.36 times higher for women and 1.58 times higher for men in the richest wealth quintile (women: AOR, 1.36; 95% CI = 1.24–1.49, P<0.001; men: AOR, 1.58; 95% CI, 1.46–171; P<0.001). Both men and women who consumed alcohol were hypertensive (men: AOR, 1.26; 95% CI, 1.21–1.31; P<0.001; women: AOR, 1.38; 95% CI, 1.23–1.55; P<0.001). Higher prevalence of hypertension was correlated with a non-vegetarian diet but results were significant only for men (AOR, 1.05; 95% CI, 1.01–1.10; P = 0.02). We also adjusted estimates for factors like religion and caste but did not find any significant results, except for Muslim women. Muslim women had greater odds of hypertension (AOR, 1.22; 95% CI, 1.14–1.30; P<0.001) compared to their other religious counterparts, but religious affiliations of men showed no significant association with hypertension.

## Discussion

This study is one of the novel attempts to assess the prevalence of hypertension and its sex-specific risk factors using the nationally representative data of NFHS 2015–2016. Additionally, the present study has also mapped the prevalence of hypertension at the state level by sex. Findings of the present study highlighted that prevalence of hypertension by all selected background characteristics is much higher for men compared to women in India. Besides this sex differences in the prevalence of hypertension we found that risk of hypertension between sexes differs by socio-economic, demographic and lifestyle factors. These results are supported by previous studies [17,18,21,25,28].

This study showed the prevalence of hypertension increases with an increase in age which is supported by previous literature [9,29–31]. The higher prevalence of hypertension among higher age groups may be attributed to a high workload and lack of physical activity. Also, at older ages, aorta and arteries walls get stiffened and this contributes to raised B.P [27]. Besides, young (15–24 years) men and women also showed considerable prevalence for hypertension in our study. But the risk of hypertension is significantly high for women at older ages although the prevalence remains low compared to men in the same age-group. Studies have documented that at old ages after menopause women have higher levels of BP than in men [32]. The higher prevalence of hypertension among men and women in urban areas indicates the harmful effect of busy lifestyles, reduced physical activity, and stressful conditions among urban residents. However, rural women are at higher risk of developing hypertension than their male counterparts. Awareness about healthcare, health-seeking and access to quality health services among women, specifically rural women, are still very limited in India [33,34]. Similarly, the prevalence and risk of high BP was higher among higher educated men possibly because they mainly work as professional/clerical and may lack intensive physical activity, have a sedentary lifestyle, and might eat food containing more fat [21] whereas we discovered that higher educated women had a better understanding of health care thus lower prevalence and risk of hypertension as documented in a previous study [35]. Previous studies also showed hypertension is higher among literate people as compared with illiterate [31,36]. The higher prevalence of hypertension among Muslims women compared to other religions largely linked to the cultural belief and influence on dietary patterns (vegetable or meat consumption). Studies showed non-vegetarian food are significantly associated with hypertension, while another study showed that a vegetarian diet is protective against hypertension [14,15]. In our study, we further found a higher prevalence of hypertension clustered among the richest as compared to the poorest sections, and this finding is supported by previous studies [21,31,37]. Also, a higher prevalence of hypertension among married samples might be explained by workload, increase in responsibility and giving less time on healthy activities like regular exercise [21,28]. However, an another study showed married person has less odd of being hypertensive compared with single and divorced or widow [31]. Men from both the richest wealth quintile and married category are more at risk of hypertension than female of the same categories.

Frequency of drinking alcohol, specifically everyday consumption of alcohol found to be significantly associated with a higher risk of hypertension in both sexes. Many pieces of the literature confirmed our findings that alcohol consumption is significantly associated with a high prevalence of hypertension [19,21,31,35]. However, results reveal that women who drink alcohol are more likely to be hypertensive. Professionals/Clerical men and women showed a higher prevalence of hypertension compared to not working. Professionals have lesser opportunities for physical movement and have sedentary lifestyles whereas agriculture and the production worker have less exposure to a sedentary lifestyle and perform intensive physical activity [9,36]. WHO also advocated for a workplace based wellness program to control hypertension [38]. Diabetic men and women showed a higher prevalence of hypertension, but diabetic women are at greater risk of being hypertensive. Unhealthy food habits, a sedentary lifestyle, and less physical activity might be the possible reason for diabetes which is also important risk factors for hypertension. The previous study also presented similar findings [37,39,40]. Hypertension is a major threat in north and north-eastern states followed by some southern states, except for Kerala. A previous study documented that high intake of salt, tobacco and alcohol consumption could be a possible reason for higher BP in north and north-eastern states [21,41,42]. The state-level differences in prevalence and risk of hypertension are linked to the diversity in cultural and dietary practices across states [43].

Although the study puts forth some of the vital information regarding hypertension, it has some limitations like the study findings are limited to specific age-groups only; for men 15–54 years and women 15–49 years. Also, it does not consider other risk factors such as regular exercise, genetic factors, obesity, stress, anxiety, and lipid profile which might be reasons for the high prevalence of hypertension among young adults. Besides, this study used cross-sectional data which could not explain the temporal relationship between the outcome variables and the explanatory variables. Hierarchical modelling technique could better discriminate and show plausible regional differences.

## Conclusion

This study attempted to fill an important gap in the literature on hypertension at the national and state level. As results suggest a substantial sex differences in the risk factors therefore, a sex-specific health care intervention might reduce this progressive disease. Besides balanced lifestyles and a healthy diet, workplace based wellness program for professionals, early screening of hypertension, raising awareness among the young generation and women from deprived sections like rural areas or minority communities who have little knowledge on health education or limited access to healthcare could play an important role to prevent hypertension in India. Thus, focusing on the prevalence and risk factors across sex is important for formulating robust health programs and policies for control of hypertension.

## Supporting information

S1 File (XLSX)
